# The aged brain: genesis and fate of residual progenitor cells in the subventricular zone

**DOI:** 10.3389/fncel.2015.00365

**Published:** 2015-09-24

**Authors:** Vivian Capilla-Gonzalez, Vicente Herranz-Pérez, Jose Manuel García-Verdugo

**Affiliations:** ^1^Laboratory of Comparative Neurobiology, Department of Cell Biology, Instituto Cavanilles de Biodiversidad y Biología Evolutiva, University of Valencia, CIBERNEDValencia, Spain; ^2^Department of Stem Cells, Andalusian Center for Molecular Biology and Regenerative MedicineSeville, Spain; ^3^Multiple Sclerosis and Neuroregeneration Mixed Unit, IIS Hospital La FeValencia, Spain

**Keywords:** neural stem cells, subventricular zone, rostral migratory stream, neurogenesis, oligodendrogenesis, cell migration, aging

## Abstract

Neural stem cells (NSCs) persist in the adult mammalian brain through life. The subventricular zone (SVZ) is the largest source of stem cells in the nervous system, and continuously generates new neuronal and glial cells involved in brain regeneration. During aging, the germinal potential of the SVZ suffers a widespread decline, but the causes of this turn down are not fully understood. This review provides a compilation of the current knowledge about the age-related changes in the NSC population, as well as the fate of the newly generated cells in the aged brain. It is known that the neurogenic capacity is clearly disrupted during aging, while the production of oligodendroglial cells is not compromised. Interestingly, the human brain seems to primarily preserve the ability to produce new oligodendrocytes instead of neurons, which could be related to the development of neurological disorders. Further studies in this matter are required to improve our understanding and the current strategies for fighting neurological diseases associated with senescence.

## Introduction

Neural stem cells (NSCs) persist in two specific regions of the adult mammalian brain: the dentate gyrus of the hippocampus, and the subventricular zone (SVZ) of the lateral ventricles (Doetsch et al., 1997; Seri et al., 2001; Garcia-Verdugo et al., 2002; Alvarez-Buylla and Lim, 2004; Quinones-Hinojosa et al., 2006). In both regions, NSCs are identified as a subpopulation of astrocytes that are able to produce the main lineages of the central nervous system (CNS), i.e., neurons, oligodendrocytes, and astrocytes (Doetsch et al., 1999a; Seri et al., 2001; Alvarez-Buylla and Garcia-Verdugo, 2002; Abrous et al., 2005; Ming and Song, 2005; Menn et al., 2006; Ihrie and Alvarez-Buylla, 2008; van den Berge et al., 2010). The production of new cells can be modulated by multiple extrinsic factors, such as an enriched environment, physical activity, stress, exposure to toxics, or drugs (van Praag et al., 1999; Brown et al., 2003; Capilla-Gonzalez et al., 2010; Ivy et al., 2010; Capilla-Gonzalez and Hernandez-Rabaza, 2011; Korosi et al., 2012), and intrinsic factors, such as growth factors, hormones, or gender (Cameron and Gould, 1994; Ohshima et al., 1996; Hirota et al., 2007). In this context, aging acts as an intrinsic factor that affects the germinal potential of the SVZ (Maslov et al., 2004; Sanai et al., 2004, 2011; Luo et al., 2006; Bouab et al., 2011; Guerrero-Cazares et al., 2011; McGinn et al., 2012; Capilla-Gonzalez et al., 2014a). Concretely, the production of new neurons is reduced with age, while the generation of oligodendroglial cells is not compromised (Bergmann et al., 2012; Capilla-Gonzalez et al., 2013). These modifications on neurogenesis may be associated with aging diseases. Here, we provide a compilation of the current knowledge about the age-related changes in the NSCs population, as well as the fate of the newly generated cells in the aged brain.

## The Subventricular Zone: A Principal Reservoir of NSCs

**Figure 1:**
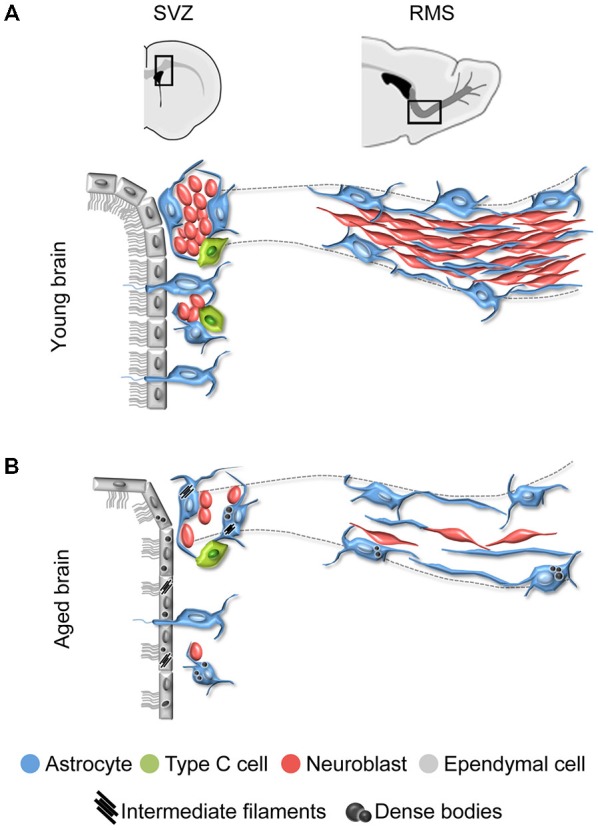
**Schematic representation of the subventricular zone (SVZ) and rostral migratory stream (RMS) in the young and aged rodent brain. (A)** In the young brain, ependymal cells with cubical morphology integrate the barrier that separates the SVZ neurogenic cells from the lateral ventricle. Neuroblasts form large chains ensheathed by gliotubes of astrocytes. Thus, neuroblasts migrate through these migratory structures, which emerge from the SVZ and coalesce into the RMS that ends in the olfactory bulb (OB). **(B)** During aging, ependymal cells are flattened and their cilia scatter. Both ependymal cells and astrocytes accumulate dense bodies and intermediate filaments in their cytoplasm. There is a decrease in the number of neural stem cells (NSCs) identified as astrocytes contacting the ventricle, intermediate progenitor cells, and neuroblasts. As a result, the RMS tends to disappear in the aged brain.

The SVZ is the main neurogenic niche in the adult mammalian brain. It is known that NSCs within the SVZ derive from embryonic radial glia cells (Merkle et al., 2004; Kriegstein and Alvarez-Buylla, 2009; Morrens et al., 2012; Fuentealba et al., 2015). During the final stages of development, radial glia cells retract their apical processes but preserve the ventricle contact, turning into the ependymal cells and NSCs of the future SVZ (Merkle et al., 2004; Spassky et al., 2005). In the adult brain, ependymal cells constitute the postmitotic population of cells within the SVZ (Spassky et al., 2005). They are cubical cells containing lipid droplets in their cytoplasm and displaying cilia and microvilli in their apical surface. Ependymal cells form interdigitations, tight junctions and adherens junctions with each other to separate the SVZ from the cerebrospinal fluid of the ventricle cavity. On the other hand, NSCs are identified as a subpopulation of astrocytes called B1 astrocytes that differ from another subpopulation of non-neurogenic astrocytes (B2 astrocytes) (Doetsch et al., 1997, 1999a; Han et al., 2008; Ihrie and Alvarez-Buylla, 2008; Mirzadeh et al., 2008; Gil-Perotin et al., 2009; Morrens et al., 2012). Briefly, astrocytes present bundles of intermediate filaments and light cytoplasm. B1 astrocytes are located next to the ependymal layer, displaying chromatin clumps close to the nuclear membrane, and a primary cilium in the apical surface that extends into the ventricle cavity. In contrast, B2 astrocytes do not contact the ventricle. B1 astrocytes proliferate and give rise to intermediate progenitor cells (type C cells), which have very large, irregular nuclei with frequent invaginations and many mitochondria in their cytoplasm. Subsequently, intermediate progenitor cells differentiate into neuroblasts (type A cells), which are small, elongated cells with a reduced dark cytoplasm, containing numerous ribosomes and microtubules (Doetsch et al., 1997, 1999a; Peretto et al., 1999; Ponti et al., 2013). Neuroblasts form large chains ensheathed by gliotubes of astrocytes that emerge from the SVZ to coalesce into the rostral migratory stream (RMS) (Lois et al., 1996; Peretto et al., 1997; Alvarez-Buylla and Garcia-Verdugo, 2002) (Figure 1A). Through the gliotubes, neuroblasts migrate tangentially long distance before they reach their final destination, the olfactory bulb (OB). Then, neuroblasts move radially and mature into interneurons that integrate in preexisting functional circuits (Lois and Alvarez-Buylla, 1994; Lois et al., 1996; Luskin et al., 1997; Carleton et al., 2003; Alvarez-Buylla and Lim, 2004; Imayoshi et al., 2008; Kelsch et al., 2010; Lazarini and Lledo, 2011). In rodents, most SVZ precursor cells become neuroblasts to support OB neurogenesis, while a small subpopulation of new cells migrates to periventricular areas such as the corpus callosum or striatum, where they give rise to myelinating oligodendrocytes, both in the normal brain and after demyelinating lesion (Nait-Oumesmar et al., 1999; Menn et al., 2006; Gonzalez-Perez et al., 2009; Capilla-Gonzalez et al., 2014b).

## Aging Disrupts the SVZ-RMS Axis

Aging is known to impact on the SVZ-RMS system, altering the ultrastructure and organization of its cells (Luo et al., 2006; Bouab et al., 2011; Capilla-Gonzalez et al., 2013, 2014a; Mobley et al., 2013). Reports have shown that the aged SVZ mostly retains ependymal cells and astrocytes. The intermediate progenitor cells are rarely found, and neuroblasts appear isolated or forming small chains. Consequently, a reduction in the neuroblast population is also observed along the RMS, which tends to disappear with age (Figure 1B). All these age-related changes are the consequence of a reduced stem cell activity (Enwere et al., 2004; Maslov et al., 2004; Luo et al., 2006; Molofsky et al., 2006; Bouab et al., 2011; Conover and Shook, 2011; McGinn et al., 2012; Capilla-Gonzalez et al., 2013).

**Figure 2:**
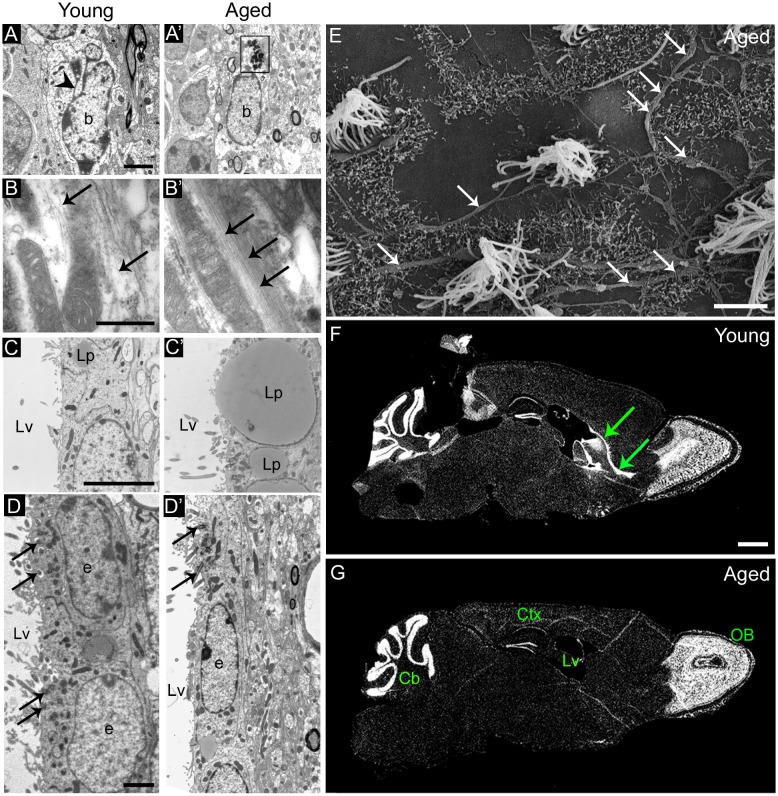
**Age-related changes in the ultrastructure of the neurogenic niches. (A,A′)** Astrocytes accumulate dense bodies (box) in their cytoplasm during aging. Scale bar: 2 micra. **(B,B′)** Detail of intermediate filaments (arrows) in astrocytic cells. Note that they are more abundant in aged cells. Scale bar: 500 nm. **(C,C′)** Detail of lipid droplets in ependymal cells, displaying a larger size during aging. Scale bar: 5 micra. **(D,D′)** Ependymal cells are flattened in the aged brain, resulting in large gaps between ciliary tufts (arrows). Scale bar: 2 micra. **(E)** Under scanning electron microscopy, whole-mount preparation of the lateral ventricle shows a deep network of axons (arrows) in the aged brain. Scale bar: 5 micra. **(F)** DAPI (4′,6-diamidino-2-phenylindole) fluorescent staining shows a remarkable RMS (arrows) from the lateral ventricle to the OB in the young brain. Scale bar: 1 mm. **(G)** Conversely, the RMS is not evident in the aged brain. Scale bar: 1 mm. b, astrocyte; e, ependymal cell; Cb, cerebellum; Ctx, cerebral cortex; Lp, lipid droplets; Lv, lateral ventricle; OB, olfactory bulb. Images **(F,G)** have been adapted with permission from Capilla-Gonzalez et al. (2013).

During aging, remaining ependymal and astrocytic cells accumulate dense bodies and intermediate filaments in their cytoplasm (Figures 2A,A′,B,B′), resembling reactive cells (Bouab et al., 2011; Capilla-Gonzalez et al., 2014a). The acquisition of a reactive phenotype in astrocytes may imply a reduction in their stemness. In fact, most of the astrocytic cells found in the aged SVZ were identified as non-neurogenic astrocytes, since they showed a lack of ventricular contact (Capilla-Gonzalez et al., 2014a). Thus, the major characteristic of aging is the reduction in proliferation that occurs in the germinal niche. Furthermore, ependymal cells in the aged SVZ present larger lipid droplets than those from young mice (Figure 2C,C′), as well as they are more flattened, which results in more dispersed cilia tufts (Luo et al., 2008; Bouab et al., 2011; Capilla-Gonzalez et al., 2014a) (Figure 2D,D′). Reports have indicated that the network of axons presented in the ventricle surface can influence the morphology of the ependymal cells. As consequence, changes in this axonal network during aging may result in the flattening of the ependymal layer. Although it has been found that the network of axons presented in the ventricle surface increases with age (Lorez and Richards, 1982; Capilla-Gonzalez et al., 2014a; Tong et al., 2014) (Figure 2E), its role in modifying ependymal cell morphology needs to be clarified. Ependymal cilia are required for normal cerebrospinal fluid flow that allows neuroblast migration based on guidance cues (Sawamoto et al., 2006; Mirzadeh et al., 2010; Young et al., 2012). Thus, the age-related changes in ependymal cilia could contribute to the reduced migration observed in old mice. In line with this idea, a similar cilia organization was observed in mice exposed to the environmental toxic N-ethyl-N-nitrosourea (ENU). Under scanning electron microscopy, the ventricle surface of these animals displayed a disorganized cilia orientation and frequent patches devoid of cilia following ENU-exposure. This ependymal ciliary dysfunction was associated with declined incorporation of SVZ-derived neuroblasts to the OB and a subsequent impairment in odor discrimination (Capilla-Gonzalez et al., 2010, 2012). Another structure that plays an important role in the adult SVZ are fractones, which are composed of ubiquitous extracellular matrix components, including heparin sulfate proteoglycans such as perlecan and agrin. Fractones can regulate neurogenesis by capturing different growth factors (Douet et al., 2012, 2013). It has been reported that aging gradually affects the number, size, and composition of these structures suggesting that, through their interaction with NSCs, fractons could be related to the loss of neurogenesis during aging (Kerever et al., 2015). According to this, changes in fractone ultrastructure have been described in an experimental obstruction model of hydrocephalus in mice, which showed decreased NSCs proliferation in the SVZ (Campos-Ordoñez et al., 2014). Future studies will provide a more comprehensive understanding on the function of fractones in the neurogenic niche. The direct consequence of aging impact on the SVZ neurogenic niche is the progressive reduction of migrating neuroblasts toward the OB (Figures 2F,G). Indeed, several studies reported how RMS is notably reduced in elderly rodent (Bouab et al., 2011; Capilla-Gonzalez et al., 2013), resulting in a severe disruption of the SVZ-RMS axis.

## Quiescence of the NSCs During Aging

Besides the decline in proliferative and neurogenic capacities of the SVZ during aging, NSCs are still found in the aged brain. However, their mitotic activity is a matter of controversy. While some studies suggest that NSCs are highly proliferative during aging (Stoll et al., 2011; Shook et al., 2012), increasing evidences indicate that the remaining actively proliferating NSCs decrease over time (Ahlenius et al., 2009; Lugert et al., 2010; Bouab et al., 2011; Encinas et al., 2011; Walter et al., 2011; Encinas and Sierra, 2012; Capilla-Gonzalez et al., 2014a). The discrepancies found in the literature could be due to the use of different strains of mice and different strategies in the analysis of proliferating cells (Schauwecker, 2006; Leuner et al., 2009; Waldron et al., 2010; Tatar et al., 2013). Most reports rely on the use of immunostaining to draw their conclusions, but aging can alter the molecular patterns expressed by the cells (McGinn et al., 2012). For instance, doublecortin (DCX) and polysialylated neuronal adhesion molecule NCAM (PSA-NCAM) expression have been used as markers of immature neurons, but they have also been presented in some populations of mature neurons, where are related to structural plasticity (Nacher et al., 2001; Bonfanti, 2006; Bloch et al., 2011). Hence, techniques based on non-molecular cues, such as retroviral-labeling or electron microscopy, could more accurately identify the nature of dividing cells. After a detailed ultrastructural analysis, a recent study revealed that most astrocytes remaining in the aged SVZ pertain to the pool of non-neurogenic astrocytes, since they did not present contact with the ventricle. In addition, the presence of abundant dense bodies and intermediate filaments in these astrocytes indicate that they acquired a reactive phenotype during aging (Capilla-Gonzalez et al., 2014a). This new phenotype may suggest that astrocytes lose their stemness over time. The proliferative ability of remaining NSCs in the aged SVZ was further assessed by incorporation of tritiated thymidine (^3^H-thymidine) and results revealed that they were able to proliferate. However, aged NSCs presented a strong intensity of the radioactive marker 6 weeks after ^3^H-thymidine injection, suggesting that they proliferate less frequently than those from the young brain, where the labeling was more diluted (Capilla-Gonzalez et al., 2014a). These data indicate that NSCs within the aged SVZ may differentiate over time, transforming into non-neurogenic astrocytes and contributing to the declined proliferation observed with age. A similar model was proposed in the aged dentate gyrus, where NSCs tend to disappear by converting into mature astrocytes (Encinas et al., 2011). At this time, further analyses are required to fully clarify this issue.

## Age-Related Changes in the Fate of Newly Generated Cells

**Figure 3:**
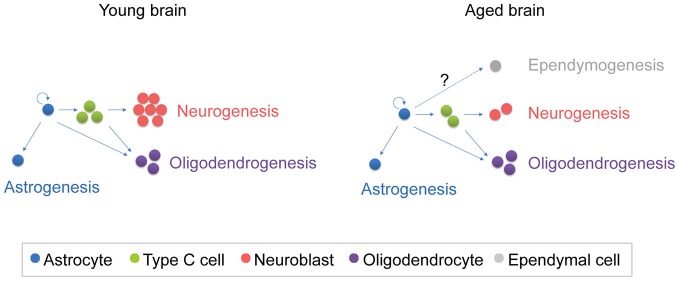
**Schematic representation of the fate of newly generated cells in the young and aged SVZ.** In the young SVZ, an important number of NSCs differentiate into neurons, while they generate oligodendrocytes and astrocytes to a lesser extent. Aging alters the balance between neurogenesis and gliogenesis. As consequence, neurogenesis is reduced in the aged SVZ, while oligodrendrogenesis is maintained. It is still under debate whether ependymogenesis occurs in the aged SVZ.

In addition to changes in the proliferation rate, the fate of the newly generated cells is modified during aging, altering the balance between neurogenesis and gliogenesis (Luo et al., 2008; Capilla-Gonzalez et al., 2013, 2014a) (Figure 3).

### Neurogenesis

The replacement of old neurons in the OB is still active in the aged brain, but it occurs to a lesser rate compared to young animals (Enwere et al., 2004; Bouab et al., 2011; Capilla-Gonzalez et al., 2013; Mobley et al., 2013). Using different techniques, previous studies have associated the alteration of the SVZ niche with an impaired neuroblast migration. For instance, localized radiation of the lateral ventricles reduces the population of precursor cells and impedes the incorporation of SVZ-derived cells into the OB (Lazarini et al., 2009; Achanta et al., 2012; Capilla-Gonzalez et al., 2014b). Similarly, the exposure to chemical agents interfering with the DNA of proliferating cells, such as ENU or cytosine-beta-D-arabinofuranoside, depleted the highly proliferative cells within the SVZ and diminished the population of migrating neuroblasts (Doetsch et al., 1999b; Capilla-Gonzalez et al., 2010, 2012). During aging, the number of active NSCs is also decreased and the production of neuroblasts declines. Hence, the impact of aging in the SVZ could be considered a cause of the reduced incorporation of new immature neurons into the OB.

### Gliogenesis

Glial cells constitute the most important cellular component of the SVZ niche. Typically, glial cells have been considered as support cells, but they also play other important roles, such as those related to the regulation of cerebral blood flow, the synaptic transmission, maintenance of cerebral metabolism, and inflammatory reactivity after injury (Sofroniew and Vinters, 2010). Astroglial cells, oligodendroglial cells, and ependymal cells are the main glial cells in the adult germinal niche.

#### Astroglial Cells

Astrocytes are the most important glial cell subtype in the SVZ (Morrens et al., 2012). Apart of their role as NSCs, astrocytes play other functions, such as supporting neuroblast migration, synaptic integration, and functional maturation of newborn neurons (Hatten et al., 1991; Song et al., 2002). Astrocytes have long been considered a homogeneous population in different brain regions based on their electrophysiological properties, markers expression, and morphology. Nonetheless, increasing evidence supports a spatiotemporal heterogeneity of astroglial populations in the brain and similarly, different spatial and temporal programs appear to diversify the progeny of SVZ astrocytes and adult NSCs (reviewed in Bayraktar et al., 2015). On the other hand, astrogenesis is essential to support the NSC population, but also to generate new astrocytes in response to brain injuries, such as stroke or traumatic lesions (Saha et al., 2013; Abeysinghe et al., 2014; Susarla et al., 2014). However, aging reduces the number of astrocytes present in the SVZ and alters the ultrastructure of these cells, i.e., increasing the presence of intermediate filaments and dense bodies in the cytoplasm (Capilla-Gonzalez et al., 2014a). It has been reported that these changes can affect the neurogenic ability of NSCs (Lim et al., 2000; Shen et al., 2008; Tavazoie et al., 2008; Kazanis et al., 2010; Ihrie and Alvarez-Buylla, 2011), thus astrogenesis property could be also compromised.

#### Oligodendroglial Cells

Oligodendrocytes have gained great significance in the last decade. This glial subtype corresponds to the myelinating cells of the CNS and is beneficial for the correct function of other neural cells. Following myelin damage, NSCs are able to produce new oligodendrocytes that participate in tissue regeneration (Nait-Oumesmar et al., 1999; Jablonska et al., 2010; Gonzalez-Perez and Alvarez-Buylla, 2011; Capilla-Gonzalez et al., 2014b). Contrary to the decline observed in OB neurogenesis during aging, increasing evidence suggests that oligodendrogenesis is maintained in the aged brain. First, SVZ NSCs of young and middle-aged mice were found to present similar ability to produce oligodendrocytes *in vitro* when they were differentiated in absence of exogenous growth factors (Bouab et al., 2011). Second, the few new cells generated in the aged mouse brain seems to change from neuronal to oligodendroglial fate in the SVZ-OB system, as revealed their tracking using different exogenous markers for dividing cells, i.e., 5-bromo-2′-deoxyuridine (BrdU) and ^3^H-thymidine (Capilla-Gonzalez et al., 2013). This age-related phenomenon has also been observed in other regions of the CNS, such as the spinal cord and neocortex of rodents (Levison et al., 1999; Lasiene et al., 2009), and the fornix of monkeys (Peters et al., 2010). The enhancement of the oligodendroglial fate with age is likely associated with a regeneration of myelin.

#### Ependymal Cells

The role of the ependymal cells in the process of neurogenesis has been controversial (Johansson et al., 1999; Spassky et al., 2005; Del Carmen Gómez-Roldán et al., 2008; Gleason et al., 2008). Although the non-neurogenic properties of the ependymal cells in the healthy brain are commonly accepted, Luo et al. (2008) suggested that ependymogenesis occurs during aging. According to this study, B1 astrocytes modify their traditional B-C-A path to generate new ependymal cells in the aged SVZ. By tracking labeled astrocytes with BrdU, it was observed that astrocytes incorporated into the ependymal layer and expressed antigenic and morphological characteristics of ependymal cells 6 weeks after BrdU administration. The new ependymal-like cells exhibited a loss of apical processes and formed adherens junctions with neighboring ependymal cells (Luo et al., 2008). This ependymal replacement was suggested to respond to damages in the integrity of the ependymal layer due to changes in the ventricle cavity (Luo et al., 2006; Conover and Shook, 2011; Shook et al., 2014). More recently, other study used ^3^H-thymidine to track astrocytes in the aged brain, but authors failed in finding astrocytes integrated into the ependymal layer that had transformed into ependymal cells (Capilla-Gonzalez et al., 2014a). In contrast, they observed that ependymal cells accumulated intermediate filaments in their cytoplasm, resembling the ependymal-like cells described by Luo et al. (2008). Supporting previous studies (Capela and Temple, 2002; Spassky et al., 2005; Young et al., 2012), authors associated these ultrastructural changes with a reactive phenotype gained by the aged cells and ruled out the possibility of the existence of proliferative ependymal cells or newly generated ependymal cells in the aged SVZ (Capilla-Gonzalez et al., 2014a). Further studies are needed to investigate the specific mechanisms altered by aging in each cell type population.

## Factors Modulating the Aged Neurogenic Niche

As mentioned above, the different cellular components of the SVZ interact with each other and with their microenvironment to regulate the neurogenic process (Lim et al., 2000; Shen et al., 2008; Tavazoie et al., 2008; Kazanis et al., 2010; Ihrie and Alvarez-Buylla, 2011; Girard et al., 2014; Capilla-Gonzalez et al., 2015). For instance, gliogenesis is induced by the bone morphogenetic protein (BMP) expression in SVZ astrocytes, while neurogenesis is promoted by Noggin, which is expressed in ependymal cells (Lim et al., 2000; Mekki-Dauriac et al., 2002; Bilican et al., 2008). Thus, the balance between neurogenesis and gliogenesis in the germinal niche is controlled by SVZ cells. Based on this observation, the modifications found in the population of astrocytes and ependymal cells during aging (Bouab et al., 2011; Capilla-Gonzalez et al., 2014a) may affect the BMP-noggin signaling, altering cell production. Other proteins, as the cellular prion protein (PrPc) and N-cadherin, have also been involved in the regulation of new cells’ fate during aging (Williams et al., 2004; Yagita et al., 2009; Bribian et al., 2012). It is known that PrPc expression is reduced during aging (Williams et al., 2004) and its suppression increases the proliferation and differentiation of oligodendrocytes (Bribian et al., 2012). Similarly, N-cadherin regulates the differentiation of glial cells in the SVZ and its blockage increases oligodendrocyte generation (Yagita et al., 2009). Considering that N-cadherin is expressed by neuroblasts, the loss of this cell type in the SVZ-OB system of aged mice could contribute to the production of oligodendrocytes, helping to maintain oligodendrogenesis. Finally, cytokines also play a key role in regulating the function of NSCs and can influence both the migration and fate of SVZ-derived cells (Yan et al., 2006; Pluchino et al., 2008; Kokovay et al., 2010; Gonzalez-Perez et al., 2012; Kang et al., 2012; Logan et al., 2013). However, this modulatory effect can be compromised during aging since cytokine expression changes (Werry et al., 2010; Gordon et al., 2012; Pineda et al., 2013). For instance, increased levels of the transforming growth factor beta (TGF-β) correlates with a decrease in neurogenesis by blocking the proliferation of SVZ precursor cells during aging (Buckwalter et al., 2006; Pineda et al., 2013; Daynac et al., 2014). This effect may be due to the fact that TGF-β is upregulated in the brain during aging (Doyle et al., 2010; Werry et al., 2010; Pineda et al., 2013). On the other hand, TGF-β1 administration promotes neuronal differentiation and survival in the SVZ (Mathieu et al., 2010), and it also increases the number of immature neurons after stroke (Ma et al., 2008). These discrepant results indicate that the TGF-β family members might regulate different mechanisms such as cell cycle, and neuronal and glial maturation. For instance, it has been proposed that TGF-β might be responsible for maintaining NSCs quiescence while promoting survival and differentiation of newly generated neurons (Kandasamy et al., 2014). Further studies are required to fully understand the mechanisms responsible of these age-related changes.

## Aging in the Human SVZ Niche

**Figure 4:**
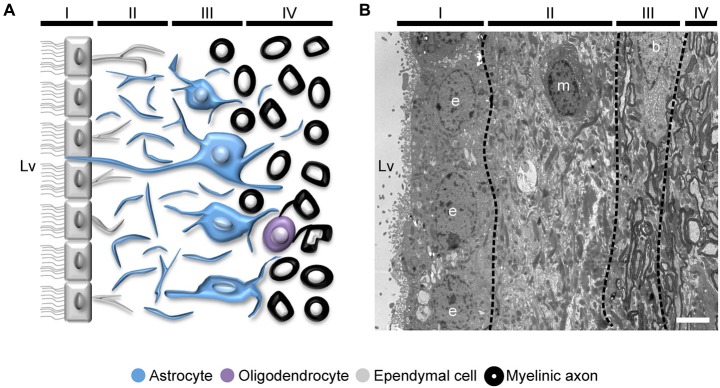
**Organization of the adult human SVZ. (A)** Diagram representing the adult human SVZ. A monolayer of ependymal cells (Layer I) separates the lateral ventricle from the SVZ. Adjacent to it, a gap or hypocellular layer is mostly composed of GFAP^+^ cellular expansions (Layer II). Next to the gap layer, the astrocyte ribbon is represented (Layer III), continued by a transition zone to the brain parenchyma (Layer IV). **(B)** Electron microscopy coronal image of the human SVZ obtained from a 53-year-old female donor. Note the typical organization of this human neurogenic niche (Layers I to IV). b, astrocyte; e, ependymal cell; m, microglia. Scale bar: 4 μm.

The organization of the adult human SVZ shows some divergences from the classical SVZ described for other mammalian species. In humans, the SVZ is composed by an ependymal layer (Layer I) that is in contact with the ventricular lumen. Next to this layer, there is a gap or hypocellular layer (Layer II), which is formed during postnatal development as a consequence of neuroblast depletion in this region. It is mostly populated by GFAP immunopositive cell expansions, although ependymal cells also send basal processes into this area. Adjacent to the hypocellular layer, there is a dense ribbon of cell bodies (Layer III) that contains astrocytes with a variable morphology, and is continued by a transition region (Layer IV) with few cells and similar to the underlying brain parenchyma (Figure 4). The human SVZ also acts as a NSCs niche capable of generating new neurons (Quinones-Hinojosa et al., 2006). During fetal and pediatric stages, SVZ-derived neuroblasts migrate via RMS into the OB (Sanai et al., 2004, 2011; van den Berge et al., 2010; Guerrero-Cazares et al., 2011). However, when the migration of neuroblasts to the OB was being studied in infants, it was unexpectedly found that there is another major migratory pathway of immature neurons destined for the prefrontal cortex (Sanai et al., 2011), suggesting that OB neurogenesis is less relevant in the human brain. In fact, the incorporation of new neurons into the human OB is nearly extinct by adulthood, as revealed by the measurement of ^14^C concentrations in the genomic DNA of these cells, which corresponded to the levels of atmospheric ^14^C at the time of birth of the examined individuals (Bergmann et al., 2012). Using the same birth dating approach, a recent study demonstrated that there is a postnatal cell turnover in the striatum of adult humans. This was corroborated by the incorporation of thymidine analog iododeoxyuridine (IdU) in striatal cells of cancer patients subjected to radiosensitization. Assessment of the expression of specific markers led these investigators to conclude that, new cells in the striatum correspond to neuronal and oligodendrocyte lineage cells (Ernst et al., 2014). Although authors suggested that these neurons likely derive from the SVZ, other origins cannot be excluded. In this regard, the production of new neurons in the adult human SVZ is still subject to debate. Most studies point to a dramatic decrease in DCX positive cells in the RMS and the OB from fetal to adult stages (Sanai et al., 2004; Wang et al., 2011). Moreover, other study demonstrated that most of the newly generated cells during adulthood correspond to non-neuronal cells, such as oligodendrocytes (Bergmann et al., 2012), suggesting that the oligodendrogenic process acquires more significance in the human brain.

Working on the human brain entails important difficulties due to the great variability present in samples (e.g., age, genetics, lifestyle…) and in their preservation quality. Discordant results among different studies could, thereby, be attributed to these causes. Therefore, it is crucial to further investigate the unique key features of adult human NSCs, which could lead us to a better understanding of neurodevelopmental and neurodegenerative pathologies.

## Concluding Remarks and Future Perspectives

Several studies on aging have established that the neurogenic niches become severely disrupted with age. The number of NSCs within the SVZ decreases over time and the generation and fate of newly generated cells is altered. Specifically, the production of neurons decreases during aging, while the generation of oligodendroglial cells seems to be preserved in the aged brain. This preservation of oligodendrogenesis in the adult brain could be crucial for the maintaining of brain functions, primarily in humans, where the production of new neurons is less relevant. On the other hand, the fact that oligodendrogenesis is unaltered over neurogenesis during aging, could indicate the importance of myelin maintenance in the aged brain, probably preventing degenerative diseases. Thus, a thorough knowledge of the events occurring during senescence becomes essential to understand the development of neurological diseases. Currently, the consequences of aging are clearly determined, but the real causes of the age-related changes are still unknown. Future studies need to be re-orientated in order to clarify this issue. The new information may potentially benefit to develop future therapeutic strategies helping to preserve the neurogenic niche, as well as its ability to participate in tissue regeneration.

## Conflict of Interest Statement

The authors declare that the research was conducted in the absence of any commercial or financial relationships that could be construed as a potential conflict of interest.
